# *BjuB*.*CYP79F1* Regulates Synthesis of Propyl Fraction of Aliphatic Glucosinolates in Oilseed Mustard *Brassica juncea*: Functional Validation through Genetic and Transgenic Approaches

**DOI:** 10.1371/journal.pone.0150060

**Published:** 2016-02-26

**Authors:** Manisha Sharma, Arundhati Mukhopadhyay, Vibha Gupta, Deepak Pental, Akshay K. Pradhan

**Affiliations:** 1 Department of Genetics, University of Delhi South Campus, New Delhi, India; 2 Centre for Genetic Manipulation of Crop Plants, University of Delhi South Campus, New Delhi, India; Chungnam National University, REPUBLIC OF KOREA

## Abstract

Among the different types of methionine-derived aliphatic glucosinolates (GS), sinigrin (2-propenyl), the final product in 3C GS biosynthetic pathway is considered very important as it has many pharmacological and therapeutic properties. In *Brassica* species, the candidate gene regulating synthesis of 3C GS remains ambiguous. Earlier reports of *GSL-PRO*, an ortholog of *Arabidopsis thaliana* gene At1g18500 as a probable candidate gene responsible for 3C GS biosynthesis in *B*. *napus* and *B*. *oleracea* could not be validated in *B*. *juncea* through genetic analysis. In this communication, we report the isolation and characterization of the gene *CYP79F1*, an ortholog of *A*. *thaliana* gene At1g16410 that is involved in the first step of core GS biosynthesis. The gene *CYP79F1* in *B*. *juncea* showed presence-absence polymorphism between lines Varuna that synthesizes sinigrin and Heera virtually free from sinigrin. Using this presence-absence polymorphism, *CYP79F1* was mapped to the previously mapped 3C GS QTL region (*J16Gsl4*) in the LG B4 of *B*. *juncea*. In Heera, the gene was observed to be truncated due to an insertion of a ~4.7 kb TE like element leading to the loss of function of the gene. Functional validation of the gene was carried out through both genetic and transgenic approaches. An F_2_ population segregating only for the gene *CYP79F1* and the sinigrin phenotype showed perfect co-segregation. Finally, genetic transformation of a *B*. *juncea* line (QTL-NIL *J16Gsl4*) having high seed GS but lacking sinigrin with the wild type *CYP79F1* showed the synthesis of sinigrin validating the role of *CYP79F1* in regulating the synthesis of 3C GS in *B*. *juncea*.

## Introduction

Glucosinolates (GS) are nitrogen- and sulfur-rich secondary metabolites characteristic of the order Capparales [1]. GS biosynthesis is highly complex. *Brassica* crops primarily contain methionine derived aliphatic GS (up to 95% of the total GS). Aliphatic GS can be broadly divided into propyls (three-carbon, 3C), butyls (four-carbon, 4C) and pentyls (five-carbon, 5C) on the basis of side chain length. *Brassica* species consist of various combinations of the above three types of aliphatic GS namely, 2-propenyl (commonly known as sinigrin) of 3C GS, 3-butenyl (known as gluconapin) and 2-hydroxy-3-butenyl (known as progoitrin) of 4C GS and 4-pentenyl (known as glucobrassicanapin) of 5C GS. The diploid *B*. *nigra* (BB, 2n = 16) contains 3C GS sinigrin, *B*. *oleracea* (CC, 2n = 18) contains either sinigrin or 4C GS, and *B*. *rapa* (AA, 2n = 20) contains both 4C and 5C GS. The GS profile of the three allotetraploid *Brassica* species, *B*. *juncea* (AABB, 2n = 36), *B*. *napus* (AACC, 2n = 38) and *B*. *carinata* (BBCC, 2n = 34) is a combination of the GS profiles of the progenitor diploid *Brassica* species [2]. In allotetraploid oilseed mustard (*Brassica juncea*; AABB), 3C and 4C constitute about 99% of the total aliphatic GS. Among the two gene pools in mustard—the east European and the Indian [3], cultivars belonging to the former primarily contain sinigrin of 3C GS whereas those belonging to the latter contain both sinigrin of 3C GS (~20%) and gluconapin of 4C GS (~80%) [4,5].

Among the different types of aliphatic GS, sinigrin, the final product in 3C GS pathway is considered to have great importance in human health as well as in plant defence processes. Its degradation product, allyl isothiocyanate has been shown to have bactericidal and antimicrobial activities [6] and anti-proliferative activities against liver [7] and bladder cancer [8]. Sinigrin and its degradation products suppress nitric oxide production in macrophages [9] and reduce plasma triglyceride level [10]. Due to its beneficial role, sinigrin is now even available as a nutrition supplement. In plants it is known to trigger stomatal closure by acting on K^+^ channels [11], prevent water loss and provide defence against fungal invasions [12] and bacterial pathogens [13].

Genetic analysis of 3C GS in *Brassica* species was initiated [14] in *B*. *napus* by QTL mapping. Since natural *B*. *napus* do not synthesize sinigrin of 3C GS, the experiment used a resynthesized *B*. *napus* line that contained the C genome from a wild form of *B*. *oleracea* which synthsizes sinigrin. The analysis led to the identification of a single locus controlling the genetic variation of sinigrin. A follow up work in *B*. *oleracea* [15] reported that alleles of a single locus *GSL-PRO* (*Arabidopsis thaliana* gene ID: At1g18500; *B rapa* gene code: Bra025899 belonging to MAM gene family) regulate the presence or absence of sinigrin. Further work in *B*. *oleracea* revealed the presence of two tightly linked duplicated genes, *GSL-PROa* (At1g18500) and *GSL-PROb* (At1g74040) by sequencing and were shown to map to the same position in the LG C5 [16,17]. However, the functional validity of *GSL-PRO* for the biosynthesis of sinigrin in *Brassica* species still remains to be demonstrated through genetic transformation experiments.

In *B*. *juncea*, the detailed genetic analysis of aliphatic GS was initiated [18] using an Indian type high seed GS line (Varuna) that contains sinigrin of 3C GS, gluconapin of 4C GS and traces of 5C GS (Table 1 of [18]) and identified four major QTL namely, *J2Gsl1*, *J3Gsl2*, *J9Gsl3* and *J16Gsl4* in LGs A2, A3, A9 and B4, respectively and one minor QTL, *J17Gsl5* in LG B1. Subsequent fine mapping study by candidate gene markers of aliphatic GS pathway genes and their co-segregation with the phenotypes [19] revealed that *J2Gsl1* controls the 4C GS as one homolog of *ELONG* gene mapped to this locus, *J3Gsl2* controls both 5C GS and total aliphatic GS as another homolog of *ELONG* and a homolog of *Myb28* mapped at a distance of 1.6 cM to the QTL region. *J9Gsl3* and *J17Gsl5* both control total aliphatic GS as one homolog each of *Myb28* mapped to these two QTL regions. The QTL *J16Gsl4* in the LG B4 was identified to be controlling the sinigrin fraction of 3C GS in *B*. *juncea*. Since the QTL *J16Gsl4* mapped to a chromosome of B sub-genome of *B*. *juncea*, it indicated that the 3C GS pathway in *B*. *juncea* is inherited from the B-genome progenitor. Furthermore, an attempt to map the *GSL-PRO* to QTL *J16Gsl4* region did not yield positive result as none of the two B genome specific paralogues for *GSL-PRO* identified in *B*. *juncea* could be mapped to QTL *J16Gsl4* that was implicated in regulating sinigrin biosynthesis in *B*. *napus* and *B*. *oleracea*. The two B-genome paralogues of *GSL-PRO* instead mapped to LGs B6 and B7 of *B*. *juncea* [19]. A follow up exercise of QTL mapping in *B*. *juncea* using an east European high seed GS line (Donskaja IV) that primarily synthesizes sinigrin also identified a QTL corresponding to *J16Gsl4* [20].

Since no candidate gene known to be responsible for the biosynthesis of sinigrin could be identified where the trait was shown to be mapping to the B genome of *B*. *juncea*, we concentrated on the identification of the candidate gene regulating sinigrin synthesis in this important oilseed and condiment *Brassica* species. In this communication we describe identification of the candidate gene responsible for biosynthesis of sinigrin in *B*. *juncea*. Analysis showed that *CYP79F1*, a homologue of the gene At1g16410 (named as *BjuB*.*CYP79F1*) mapped to the QTL *J16Gsl4* in LG B4 of *B*. *juncea*. Further, we confirmed the role of *BjuB*.*CYP79F1* in regulating the synthesis of sinigrin by validation through genetic and transgenic approaches in *B*. *juncea*.

## Materials and Methods

### Plant Materials used

Several genetic stocks consisting of different oilseed *Brassica* species and two mapping populations were used in the study. For gene isolation experiment, two *B*. *juncea* (AABB) lines—Varuna [a high GS Indian variety (>110 μmol g^-1^ seed with 18–20 μmol g^-1^ seed of sinigrin)] and Heera [a low GS east European line (~12 μmol g^-1^ seed and virtually free from sinigrin) (Table 1 of [18]) containing low alleles for *Myb28*, *ELONG*s and QTL *J16Gsl4* [19] were used. The study also includes one line each from *B*. *rapa* (AA) cv. YSPB-24 and *B*. *nigra* (BB) cv. IC 257 with high seed GS content. For genetic analyses and mapping, two bi-parental populations–(i) A Varuna x Heera F_1_DH mapping population (VH) consisting of 123 lines (the population was previously used in the lab to construct a high-density comparative linkage map in *B*. *juncea*) [21] and QTL mapping of seed GS [18]. (ii) An F_2_ population of 95 segregants derived from a cross between Varuna x QTL-NIL *J16Gsl4*. The line QTL-NIL *J16Gsl4* is a near isogenic line (NIL) which contains Heera allele of QTL *J16Gsl4* in the genetic background of Varuna and is high in seed GS (>110 μmol g^-1^ seed) with trace amount of sinigrin. The NIL *J16Gsl4* was developed by following a recurrent selection backcross (RSB) scheme [18] and was identified as NIL (near iso-genic line) for the QTL *J16Gsl4* through genotyping for all GS QTL/genes from a population of 785 BC_4_DH lines segregating for aliphatic GS trait [19]. Finally for the transformation experiment, two lines namely, QTL-NIL *J16Gsl4* (high seed GS) and EH-2 (an EMS induced early flowering mutant of low GS line Heera containing mutant alleles for the loci *Myb28*, *ELONG*s and QTL *J16Gsl4* as in Heera and also having comparable aliphatic seed GS profile as that of Heera) were used. We used EH-2 in place of Heera because the lab has standardized transformation protocol for EH-2.

### Cloning, sequencing and mapping of the gene

Genomic DNA was isolated from well-expanded leaves from the field grown plants following the protocol of Rogers and Bendich [22]. All PCR amplifications were carried out in a 20 μl reaction volume containing 50 ng of template DNA, 800 μM of dNTPs, 2 mM of MgCl_2_, 10 pmol each of forward and reverse primers and enzyme Taq polymerase (1 Unit, i-Taq DNA polymerase, Intron Biotechnology, Inc.). The PCR profile included initial denaturation at 94°C for 5 min followed by 36 cycles of 94°C for 30 sec, annealing at 62°C for 30 sec and 72°C for 1 min 30 sec and a final extension at 72°C for 12 min. For the purpose of sequencing, amplified fragments were cloned in the pGEM-T Easy TA cloning vector (Promega, Madison, USA). Sequencing was done by capillary electrophoresis on Applied Biosystems 3730 Genetic Analyzer. Sequencing of each sample was done in a minimum of 3 independent replicates to remove chances of any PCR related error. Sequence files were assembled and analysed using EditSeq, SeqMan and MegAlign software packages of DNAStar (DNAStar Inc.). For mapping, marker genotyping data of the mapping population were added to the existing map of *B*. *juncea* [21] using the program JoinMap version 4.0 [23]. Genome walk was performed using PCR genomic libraries based on the method described by GenomeWalker Universal Kit (Clontech Ltd.).

### Screening of BAC libraries and verification

Development of BAC libraries used in the study has been described by Sharma et al. [24]. BAC screening, identification and contig construction was performed according to the protocol followed by Sharma et al. [24].

### Sequencing of BAC and sequence analysis

Sizing of the BAC insert and Sanger sequencing of BAC was outsourced to Amplicon Express, USA. BAC sequencing by 454/Roche GS-FLX Titanium pyrosequencing and sequence assembly using GS De Novo Assembler (v 2.9) was performed at Macrogen Inc., South Korea. Annotation of the assembled contigs was performed using the *Brassica* BAC annotation pipeline (http://brassica.nbi.ac.uk/annotate.html). Transposable elements (TE) in the BAC were predicted and located by using the program RepeatMasker (http://www.repeatmasker.org/). Sequence of the BAC was aligned to the corresponding *A*. *thaliana* sequences obtained from TAIR (https://www.arabidopsis.org/) and *B*. *rapa* sequences obtained from BRAD (http://brassicadb.org/brad/) using NCBI BLAST (http://blast.ncbi.nlm.nih.gov/Blast.cgi?PAGE_TYPE=BlastSearch&PROG_DEF=blastn&BLAST_PROG_DEF=megaBlast&BLAST_SPEC=blast2seq) to analyze gene conservation.

### Reverse transcriptase (RT)-PCR

Total RNA was extracted from frozen tissues using the RNeasy Plant Mini Kit (Qiagen), complying with manufacturer’s instructions. Total RNA isolated was treated with DNase using the RNase-Free DNase Set (Qiagen) and column purified. cDNA library was synthesized by reverse transcribing 2 μg of total RNA with High Capacity cDNA Reverse Transcription Kit (Applied Biosystems, USA) in a 20 μl reaction volume using mixture of random primers and oligo-dTs in 1:1 ratio according to manufacturer’s instructions. PCR amplification was carried out in a 20 μl reaction volume containing 1 μl of cDNA, 800 μM of dNTPs, 2 mM of MgCl_2_, 10 pmol each of forward and reverse primers and 1 Unit of Taq polymerase enzyme (i-Taq DNA polymerase, Intron Biotechnology, Inc.). The RT-PCR profile included initial denaturation at 94°C for 5 min followed by 30 cycles of 94°C for 30 sec, annealing at 60°C for 30 sec and 72°C for 30 sec and a final extension at 72°C for 5 min. The PCR products were analysed by agarose gel electrophoresis. Actin gene forward primer Actin-FP (5’–GGC TCC TCT TAA CCC AAA GG– 3’) and reverse primer Actin-RP (5’–TTC TCG ATG GAA GAG CTG GT– 3’) was used as endogenous control for equal template loading.

### Genetic transformation of *B*. *juncea* and GS analysis

*Agrobacterium tumefaciens* mediated genetic transformation of *B*. *juncea* was performed using hypocotyl explants following the protocol described by Jagannath et al. [25]. Extraction and estimation of seed GS content and analysing the GS profiles was done by HPLC [20].

### Data deposition

The sequences reported in this paper are deposited in the National Center for Biotechnology Information GenBank database with accession numbers KT254222 (*BjuB*.*CYP79F1* from *B*. *juncea* cv. Varuna) and KT254223 (*BniB*.*CYP79F1* from *B*. *nigra* cv. IC 257).

## Results

### Identification and amplification of aliphatic GS genes from QTL *J16Gsl4* region of *B*. *juncea*

**Fig 1:**
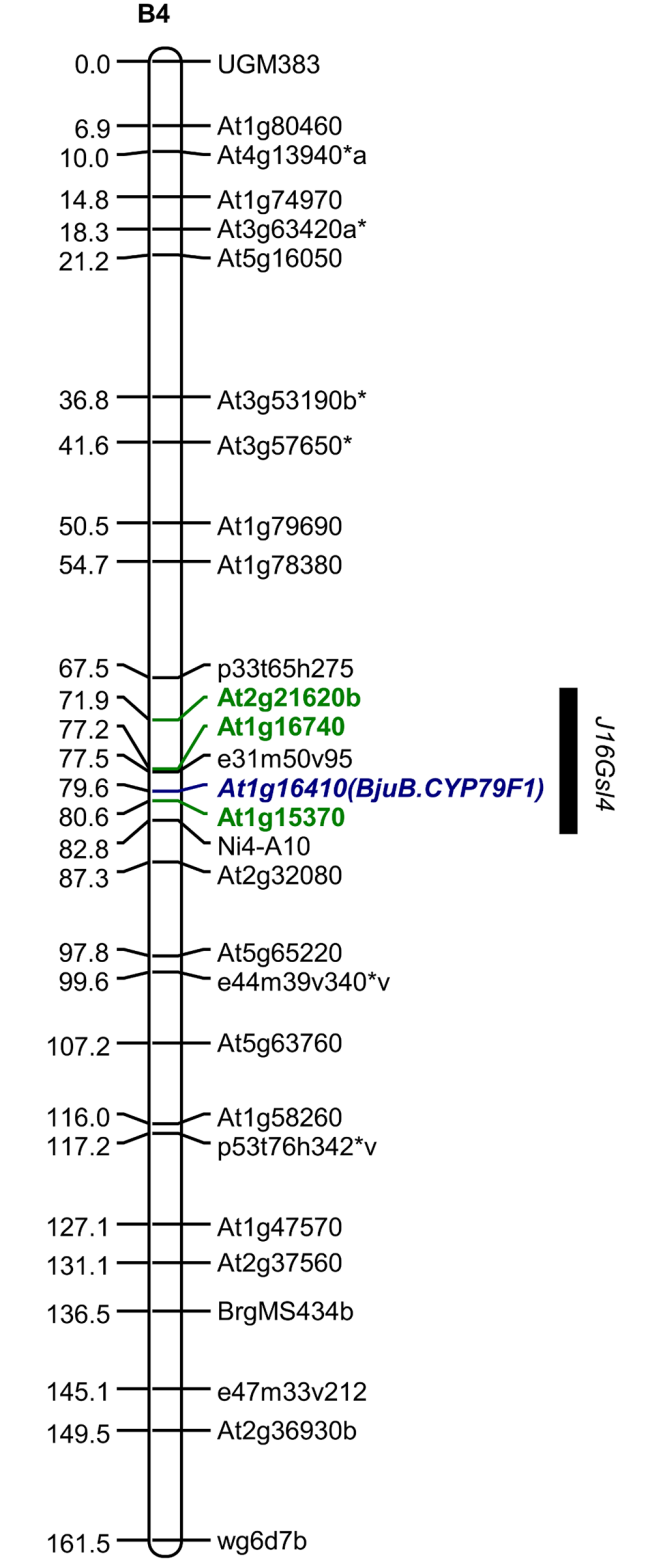
Map of LG B4 showing positions of marker At1g16410 (named as *BjuB*.*CYP79F1*) and QTL *J16Gsl4* in *B*. *juncea*.

An *in silico* search of aliphatic GS genes from 1g and 2g chromosomes of Arabidopsis ([26] (sequences downloaded from http://www.arabidopsis.org/) encompassing the syntenous region of QTL *J16Gsl4* (Fig 1) flanked by the markers At1g15370 (block A) and At2g21620 (block I) in *B*. *juncea* [21] and the corresponding regions in *B*. *rapa* ([27] (sequences downloaded from http://brassicadb.org/brad/) was undertaken. The search identified a total of 15 genes of aliphatic GS biosynthetic pathway (*GSL-PRO* not included). A total of 14 degenerate primers (wherever necessary) to accommodate the multiple copies of the genes were used (S1 Table) for amplification from two allotetraploid *B*. *juncea* (AABB) lines Varuna and Heera having contrasting phenotype for sinigrin and also from the diploid parental species *B*. *rapa* cv. YSPB-24 (AA) and *B*. *nigra* cv. IC257 (BB). None of these primer pairs showed clear-cut polymorphism between Heera and Varuna (S1 Fig). Hence, emphasis was laid for uncovering B genome specific allelic polymorphism of the genes as the QTL *J16Gsl4* regulating the sinigrin synthesis was located in the LG B4 of *B*. *juncea*. The process was initiated with the genes *CYP79F1* (At1g16410) and *CYP79F2* (At1g16400) (tandemly duplicated in Arabidopsis) involved in the first step of synthesis of core GS structure. Priority was given to these two genes because of the fact that these gene IDs (At1g16400 and At1g16410) were assumed to be located nearer to the peak LOD value marker (At1g16740) of the QTL *J16Gsl4* [18,19] (Fig 1) as revealed by the synteny information from a comparative map of *B*. *juncea* [21].

### Isolation of gene *BjuB*.*CYP79F1* from *B*. *juncea* and its mapping

**Fig 2:**
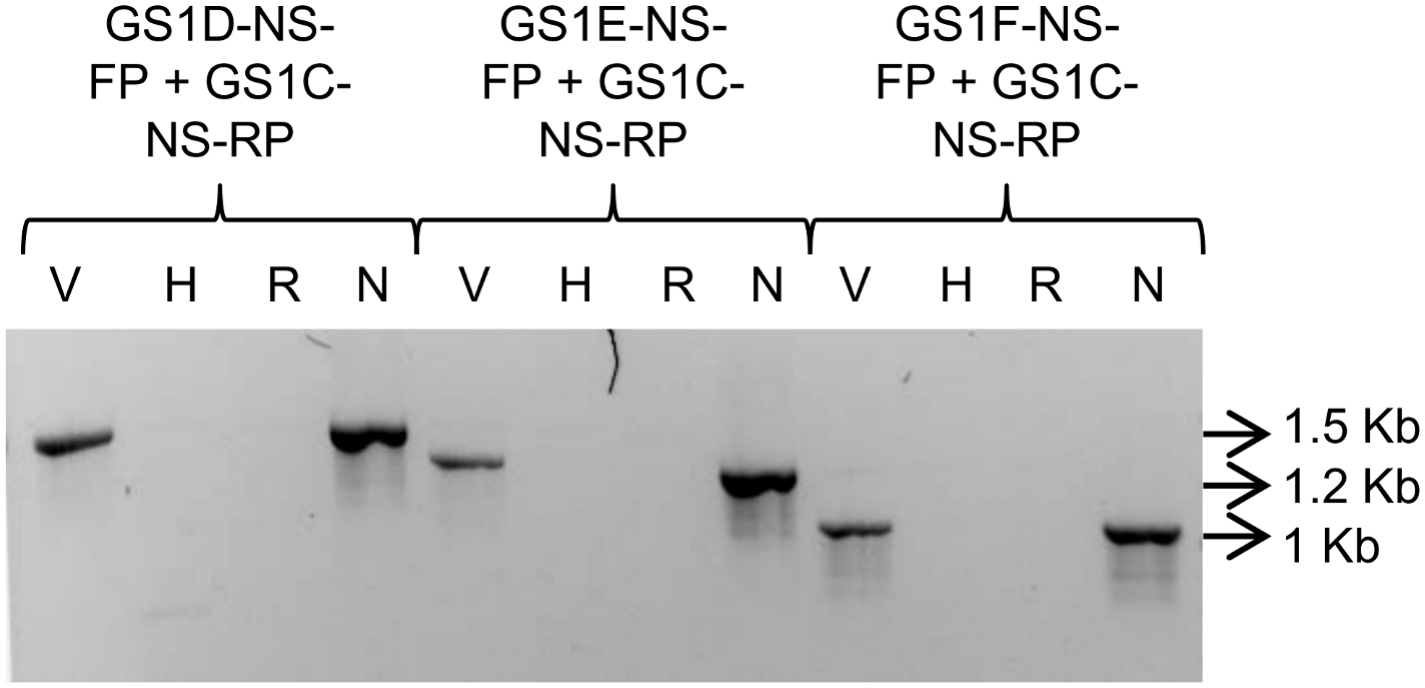
PCR amplification by three B genome specific primers (names of primers used have been given on top of the gel lanes) for *CYP79F1* from *B*. *juncea* lines Varuna (V) and Heera (H); *B*. *rapa* (R); *B*. *nigra* (N).

The initial non-polymorphic amplified products (S1 Fig: ~1700 bp from the primer pair GS1-FP and GS1-RP) from the two parental species *B*. *rapa* and *B*. *nigra* which were presumed to contain the sequences of both *CYP79F1* and *CYP79F2* were cloned into pGEM-T easy vector and sequenced. The sequences from both 5’ and 3’ ends of the cloned products revealed only one sequence variant each in *B*. *rapa* and *B*. *nigra*. Based on the sequence divergence from the deduced partial sequences of the two orthologs of *CYP79F1*/*CYP79F2* from *B*. *rapa* and *B*. *nigra*, three sets of *B*. *nigra*-specific forward primers (GS1D-NS-FP, GS1E-NS-FP and GS1F-NS-FP) and one reverse primer (GS1C-NS-RP) were designed for amplification (for detailed primer sequences see S2 Table and for the positions of the primers see S2 Fig). PCR amplification using these three sets of *B*. *nigra*-specific nested primers led to the amplification of only one band of different molecular weights by each primer. *B*. *nigra* (B genome) specificity of these three primers was confirmed by the fact that none of the primer pairs amplified any bands from *B*. *rapa* (A genome), whereas all the three primers amplified one band each from *B*. *juncea* line Varuna with the same molecular weight as that from *B*. *nigra* (Fig 2). However, no band was amplified from Heera indicating that the gene is either truncated or lost in the B genome of *B*. *juncea* line Heera.

Using one of these three primer pairs (GS1D-NS-FP and GS1C-NS-RP) showing dominant polymorphism between Varuna and Heera, the gene *CYP79F1/CYP79F2* was mapped to the existing VH map of Panjabi et al. [21] (Fig 1). It mapped to the QTL interval of QTL *J16Gsl4* indicating that the *CYP79F1/CYP79F2* could be the probable candidate gene for biosynthesis of sinigrin.

Complete sequences of the B genome orthologs of the gene *CYP79F1/ CYP79F2* from *B*. *juncea* cv. Varuna and from *B*. *nigra* cv. IC257 were derived by using a common set of nested primers and through 5’ and 3’ genome walking (See S2 Table for primer sequences). Complete genomic sequence of the gene in Varuna was observed to be 2163 bp long (S2 Fig). A data search of genome sequences of *Brassica* species revealed the presence of only *CYP79F1* in *B*. *rapa* [27], *B*. *oleracea* [28] and *B*. *napus* [29]. Alignment by BLAST search of these two deduced sequences from *B*. *nigra* and *B*. *juncea* with the *CYP79F1* from *B*. *rapa*, *B*. *oleracea* and *B*. *napus* and the two tandemly duplicated genes *CYP79F1* and *CYP79F2* from Arabidopsis revealed high similarity with *CYP79F1* of *Brassica* species than the sequences of *CYP79F1* and *CYP79F2* of Arabidopsis. Hence, the isolated genes from the B genome of *B*. *juncea* and from *B*. *nigra* were named as *BjuB*.*CYP79F1* and *BniB*.*CYP79F1*, respectively. The predicted coding region comprised three exons, as in *CYP79F1* from *A*. *thaliana* (S3 Fig). The coding sequence of *BjuB*.*CYP79F1* was compared with the orthologous sequences of the gene from *A*. *thaliana* (At1g16410 and At1g16400), *B*. *nigra* (*BniB*.*CYP79F1*, isolated in the present study) and other *Brassica* species. The gene was almost identical to *BniB*.*CYP79F1* (99.8%) and also showed high sequence identity (>93%) with *CYP79F1* of other *Brassica* species (S4 Fig).

The full length 1626 bp *BjuB*.*CYP79F1* cDNA encodes a predicted 62 kDa polypeptide of 541 amino acids. Several motifs in the deduced amino acid sequences of BjuB.CYP79F1 exhibited similarities with the conserved motifs found in cytochrome P450 enzymes: a short N-terminal signal peptide for targeting to the endoplasmic reticulum (ER), a stretch of hydrophobic amino acids constituting the transmembrane helix, which anchors the protein in the ER; a positively charged domain; a proline-rich motif and a heme binding domain towards the C-terminal [30,31]. When queried using the PredictProtein software (https://www.predictprotein.org/; [32,33]), subcellular localization of BjuB.CYP79F1 and BniB.CYP79F1 was predicted to be in the ER membrane, and amino acids 16–34 in BjuB.CYP79F1 and amino acids 14–34 in BniB.CYP79F1 were predicted to constitute the transmembrane helix (S3 Fig).

### Resolving the structural organization of *BjuB*.*CYP79F1* in Heera

Our initial PCR experiment (previous section) could not succeed in amplifying the *BjuB*.*CYP79F1* from the parent Heera. Hence, the gene organization of *BjuB*.*CYP79F1* in Heera was resolved through BAC sequencing. Initially, a sequence of 1177 bp starting from -645 to +532 bp was established by 5’genome walking which showed that the 5’ end of the gene in Heera is intact. The sequence was identical to the corresponding sequence of the gene in Varuna. Subsequently, a primer pair that would amplify -428 to +532 bp was used for screening the two BAC libraries of the Heera genome [24]. Of the four positive BAC clones identified, the clone with the biggest insert was sequenced.

**Fig 3:**
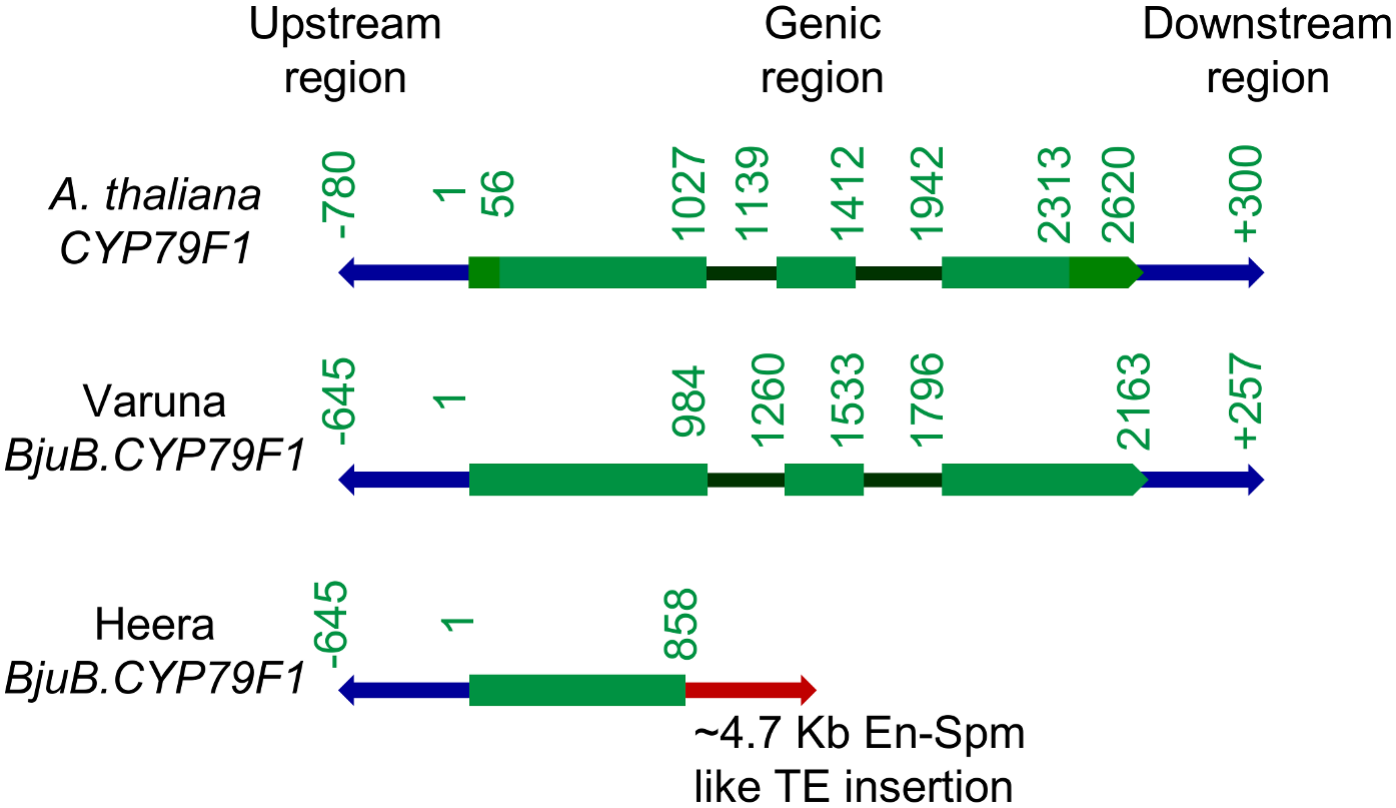
Representative gene models depicting structural organization of *CYP79F1* from Arabidopsis and two *B*. *juncea* lines Varuna and Heera. Bases 1–55 and 2313–2620 in *A*. *thaliana CYP79F1* constitute the 5’UTR and 3’UTR, respectively.

The BAC sequence data revealed the length of the insert as 94.989 kb and predicted a total of 30 genes (S3 Table). The sequence analysis also uncovered the presence of 24 transposable elements (TE) in the region. The gene *BjuB*.*CYP79F1* was observed to be truncated in the first exon with next two exons missing. The truncated first exon was completely identical up to 855 bp from the 5’ end with *B*. *rapa CYP79F1* (Bra026058) and up to 858 bp from the 5’ end with wild type *BjuB*.*CYP79F1* gene of Varuna. Following the truncation point, presence of a ~4.7 Kb long En-Spm like DNA transposon downstream of *BjuB*.*CYP79F1* was detected (Fig 3). It suggests that the insertion of the transposon might have led to exon loss, and probably caused the loss of function of *BjuB*.*CYP79F1* in Heera.

### Functional validation of *BjuB*.*CYP79F1* as the candidate gene responsible for 3C GS

#### Genetic approach

An F_2_ population of 95 individuals from a cross between Varuna and QTL-NIL *J16Gsl4* segregating for the sinigrin was used for the validation through co-segregation of the genotype and the phenotype. The scheme for the development of F_2_ population has been shown in S5 Fig. The NIL status of QTL-NIL *J16Gsl4* was initially established using 59 background markers distributed throughout the genome spanning over 18 LGs of *B*. *juncea* and two flanking foreground markers (At1g15370 and At2g21620b) (Fig 1). The genotyping data confirmed the line as QTL-NIL *J16Gsl4* as all the background markers revealed the genotype of the recurrent parent Varuna except a ~13.9 cM introgression of the QTL region of *J16Gsl4* from the donor parent Heera.

Analysis of 95 F_2_ segregants revealed a perfect co-segregation between the phenotype and the genotype wherein F_3_ seeds from 76 F_2_ segregants showed the synthesis of sinigrin ranging from 8.1–31.5 μmol g^-1^ (mean 13.7 μmol g^-1^) and the presence of ‘Varuna’ allele for *BjuB*.*CYP79F1*. The remaining 19 segregants showed no synthesis (or traces) of sinigrin and the presence of ‘Heera’ allele for *BjuB*.*CYP79F1* (S6 Fig). The segregation data conformed to the 3:1 ratio showing a single locus inheritance (Table 1). Details of the aliphatic seed GS profiles of parents and F_2_ segregants have been shown in S4 Table.

**Table 1:** Phenotypic (for sinigrin) and genotypic (for *BjuB*.*CYP79F1*) co-segregation data of a F_2_ population from a cross between QTL-NIL *J16Gsl4* line and Varuna.

Total number of segregants^1^	Observed frequency of co-segregation between phenotype and genotype^2^		χ^2^ values for 3:1 ratio^3^
	Presence	Absence	
95	76	19	1.261

^1^ F_3_ seeds obtained from F_2_ plants were analyzed as the GS trait exhibits maternal effect.

^2^ For detailed co-segregation see S6 Fig and S4 Table.

^3^ Table value of χ^2^_0.05_ at 1 df = 3.841.

**Fig 4:**
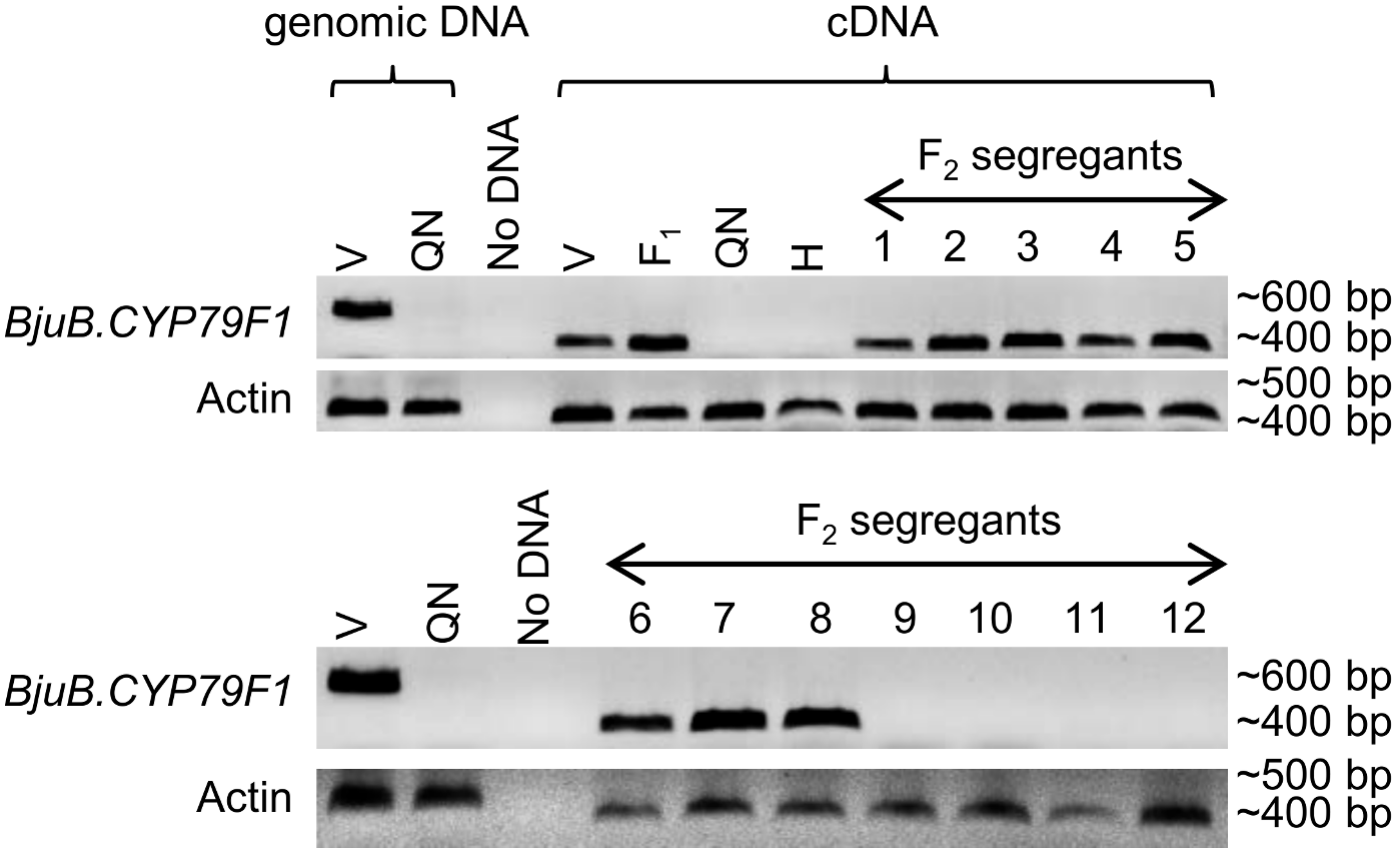
RT-PCR analysis from developing siliques showing expression of *BjuB*.*CYP79F1* in parents and F_2_ segregants derived from a cross between Varuna and QTL-NIL *J16Gsl4*. RT-PCR reactions were performed using gene specific primers for *BjuB*.*CYP79F1* (forward primer GS1G-NS-FP and reverse primer GS1B-NS-RP) and Actin (forward primer Actin-FP and reverse primer Actin-RP). Amplification from genomic DNA was done by primer pair GS1G-NS-FP and GS1B-NS-RP (S2 Table). No DNA lane was used as control. V—Varuna, QN—QTL-NIL *J16Gsl4* line, F_1_ –F_1_ between Varuna x QTL-NIL *J16Gsl4*, H—Heera, cDNA—complementary DNA, 1–8 –F_2_ segregants where *BjuB*.*CYP79F1* is present (Varuna allele), 9–12 –F_2_ segregants where *BjuB*.*CYP79F1* is absent (Heera allele).

An expression analysis was done from the developing siliques (20 days after pollination) of the F_2_ segregants through reverse transcriptase (RT) PCR. Eight randomly selected F_2_ plants that showed the presence of gene *BjuB*.*CYP79F1* (Varuna allele) and four F_2_ plants that did not (Heera allele) were chosen for the expression analysis. All the eight F_2_ segregants showing the presence of the gene *BjuB*.*CYP79F1* also showed amplification from *BjuB*.*CYP79F1* cDNA. On the other hand, the four lines that did not amplify the gene *BjuB*.*CYP79F1* did not show amplification from cDNA as well (Fig 4). This lack of transcription from the parents Heera, QTL-NIL *J16Gsl4* and four F_2_ segregants containing ‘Heera’ allele indicates that the truncated *BjuB*.*CYP79F1* of Heera is non-functional.

#### Transgenic approach

**Fig 5:**
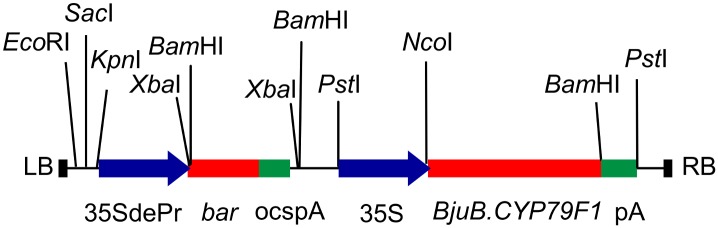
Map of T-DNA construct of *BjuB*.*CYP79F1* used for genetic transformation of *B*. *juncea*.

A gene construct for plant transformation was developed under the control of CaMV35S promoter and terminator that contained wild-type *BjuB*.*CYP79F1* from Varuna in the binary vector pPZP200 (Fig 5). The *bar* gene, conferring phosphinothricin resistance and driven by double enhancer CaMV35S promoter was used as a selectable marker. The construct was introduced into electro-competent *Agrobacterium tumefaciens* strain GV3101. Two *B*. *juncea* lines, EH-2 (low seed GS content) and QTL-NIL *J16Gsl4* (high seed GS content) both almost free from sinigrin and containing Heera allele of the gene, were used for transformation.

**Fig 6:**
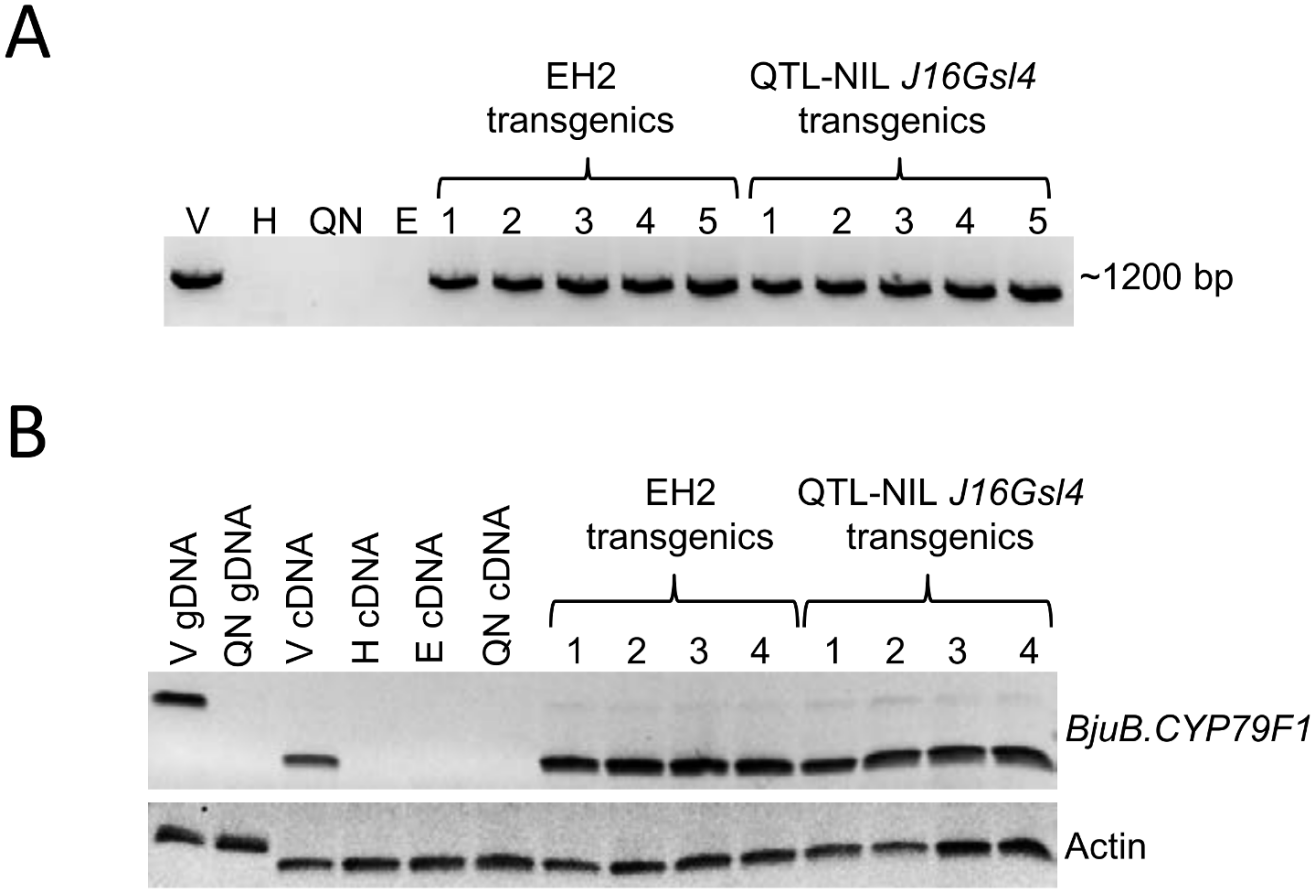
(A) Gel picture showing amplification of *BjuB*.*CYP79F1* (PCR using forward primer GS1B-NS-FP and reverse primer GS1B-NS-RP) in five T_0_ transgenics each of EH-2 and QTL-NIL *J16Gsl4*. (B) Expression analysis of *BjuB*.*CYP79F1* from leaves of T_0_ transgenic lines by RT-PCR using the same primers as in Fig 4. V—Varuna, H—Heera, E—EH-2, QN—QTL-NIL *J16Gsl4*, gDNA—genomic DNA, cDNA—complementary DNA.

A total of 37 and 211 independent putative (T_0_) transgenic plants of EH-2 and QTL-NIL *J16Gsl4*, respectively, were transferred to a containment net house during the mustard growing season. Basta (200 mg l^-1^) was sprayed 30 days after transplantation. Basta resistant plants were grown to maturity and were allowed to set seeds through selfing as well as open pollination. The presence of the transgene and its expression were confirmed by PCR amplification of the genomic DNA (Fig 6A) and by RT-PCR of leaf mRNA (Fig 6B) respectively, from randomly selected T_0_ plants of QTL-NIL *J16Gsl4* and EH-2. Control (untransformed) parental lines namely, Varuna, EH-2 and QTL-NIL *J16Gsl4* were also grown in the containment net house for comparative seed GS profile analysis.

**Fig 7:**
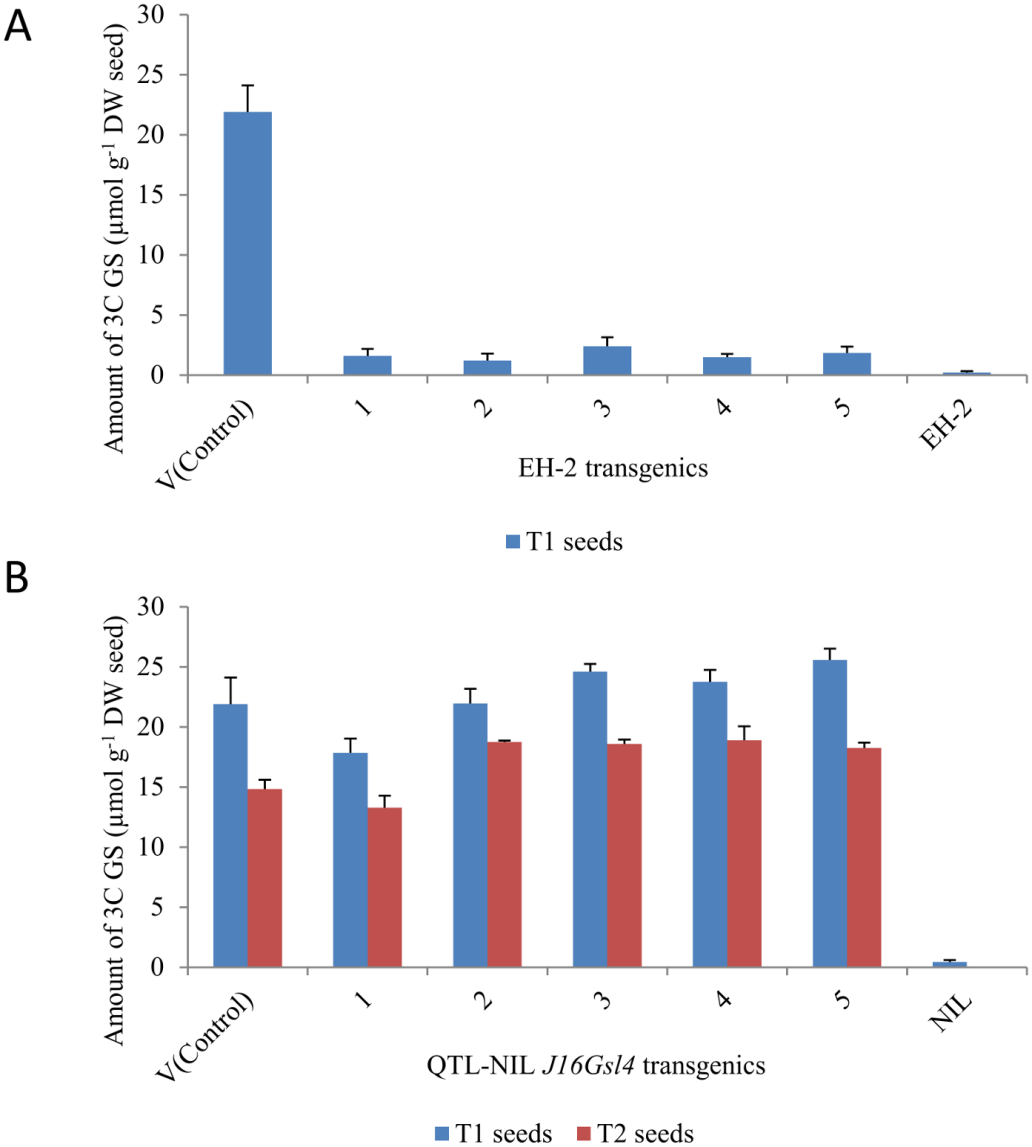
Graph showing seed 3C GS profile (T_1_ and T_2_ seeds) of five independent lines showing high sinigrin expression each from (A) EH-2 (T_1_ seeds only) and (B) QTL-NIL *J16Gsl4* containing transgene 35S:*BjuB*.*CYP79F1*.

Open-pollinated T_1_ seeds from 20 randomly selected transgenic plants each from EH-2 and QTL-NIL *J16Gsl4* along with the untransformed parental controls were analysed for total GS and profiles of aliphatic GS by HPLC. The aliphatic GS profiles of seeds from controls (with high GS) grown under containment net house showed an upward trend in the seed GS content (S5 Table) as compared to the plants grown in open field (S4 Table). We invariably, observe this trend between the field grown and net house grown plants as several environmental factors influence the GS production [2]. The EH-2 transgenic lines had low GS content conforming to the status of the wild type parent EH-2. It was also observed that none of these 20 transgenic lines showed significant increase in the sinigrin content as compared to the untransformed wild type. The range of sinigrin content varied from 0.2 to 2.4 μmol g^-1^ dry weight. It implies that even the presence of a functional copy(ies) of *BjuB*.*CYP79F1* gene did not enhance synthesis of sinigrin in the low GS EH-2 transgenics. On the other hand, the range of sinigrin content in the 20 transgenic lines of QTL-NIL *J16Gsl4* varied from 0.5 to 25.5 μmol g^-1^ dry weight wherein five transgenic lines showed sinigrin content varying from 17.8 to 25.5 μmol g^-1^ dry weight in the T_1_ seeds. Selfed T_1_ seeds of these five QTL-NIL *J16Gsl4* transgenic lines showing high sinigrin content were grown in containment in the next growing season to obtain T_2_ seeds. After Basta (200 mg l^-1^) spraying, resistant segregants of each line were grown to maturity and selfed seeds from each line were harvested separately. Analysis of open-pollinated T_2_ seeds by HPLC revealed consistency in the sinigrin content and was comparable to the sinigrin content of the wild type *B*. *juncea* line Varuna (Fig 7, S5 Table). It implies that *BjuB*.*CYP79F1* has effectively restored sinigrin biosynthesis in QTL-NIL *J16Gsl4* line, which lacked only sinigrin in its otherwise high GS profile.

## Discussion

Prior information on QTL mapping of the 3C aliphatic GS (*J16Gsl4*) to the LG B4 [18] and comparative mapping of *B*. *juncea* genome [21,34] along with the genome sequence data of both Arabidopsis and *B*. *rapa* helped in searching for the *CYP79F1* gene in the QTL region. The genome search of *B*. *rapa* (AA) [27], *B*. *oleracea* (CC) [28] and *B*. *napus* (AACC) [29] revealed the presence of only *CYP79F1* in all these *Brassica* species. The gene in the A genome of *B*. *napus* was observed to be tandemly duplicated. In the present study, we detected only *CYP79F1* (*BjuB*.*CYP79F1*) from the B genome of *B*. *juncea* and also one each from *B*. *nigra* and *B*. *rapa*. It indicated that at least two copies of *CYP79F1*, one in each homoeolog, should be present in *B*. *juncea*.

The observed truncation of the gene *BjuB*.*CYP79F1* in *B*. *juncea* line Heera (Fig 3) due to the insertion of a ~4.7 kb TE in the first exon led to the loss of function of the gene as no mRNA was detected either from the developing siliques or from the leaves (Figs 4 and 6B). It could be substantiated with the fact that Heera is a low GS line containing primarily 4C GS and only trace amount of sinigrin (3C GS). On the other hand, it was possible to isolate the full length gene of *BjuB*.*CYP79F1* from the wild type *B*. *juncea* cv. Varuna that synthesizes sinigrin. The gene was almost identical to *BniB*.*CYP79F1* (99.8%) of *B*. *nigra* and showed high sequence identity (>93%) with *CYP79F1* of other *Brassica* species. The BjuB.CYP79F1 protein was predicted to be localized on the ER membrane inside the cell. It is known that methionine derivatives with carbon side chains are synthesized inside the chloroplast [35] and are translocated across the chloroplast membrane to CYP79F1 which is anchored on the ER [31]. High level of identity and similar domain structure deduced from the sequence of BjuB.CYP79F1 with the known and characterized CYP79F1 from *A*. *thaliana* suggests that these are orthologous genes and in all probability perform similar function.

Validation of *BjuB*.*CYP79F1* (a B genome homoeolog of *CYP79F1*) as the gene responsible for regulating sinigrin biosynthesis in *B*. *juncea* was first undertaken by genotype-phenotype co-segregation analysis. All the Varuna x QTL-NIL *J16Gsl4* F_2_ segregants containing the Varuna allele of the gene (wild type) showed synthesis of sinigrin while the segregants having Heera allele did not show synthesis of sinigrin (S6 Fig).

The final validation for the gene was obtained by genetic transformation of the line QTL-NIL *J16Gsl4* with the gene construct containing wild type gene *BjuB*.*CYP79F1*. QTL-NIL *J16Gsl4* is a high GS line having truncated *BjuB*.*CYP79F1* allele from Heera and is devoid of sinigrin in the seed. The observation of the synthesis of sinigrin up to 25.5 μmol g^-1^ in the T_1_ transgenic seeds comparable to the sinigrin content of wild type line Varuna (21.9 μmol g^-1^ seed; Fig 7) is indicative of the fact that *BjuB*.*CYP79F1* is the candidate gene regulating the synthesis of sinigrin in *B*. *juncea*. Similar trend of sinigrin synthesis in T_2_ transgenics indicated the stable inheritance of the trait. Moreover, the use of QTL-NIL *J16Gsl4* that contains Heera allele for the locus *J16Gsl4* and Varuna alleles for all other loci in GS pathway indicated that no other enzyme in the pathway produced by Varuna allele is compensating for the synthesis of sinigrin. We, therefore, propose that *BjuB*.*CYP79F1* plays a major role in the conversion of homomethionine to 4-methylthiobutanaldoxime that leads to the synthesis of sinigrin according to the proposed GS pathway [1]. That some of the transgenic lines of QTL-NIL *J16Gsl4* in the study did not show increase in the sinigrin content could be attributed to position effect causing low expression of the transgene in these lines. This type of gene expression variation is of general occurrence in transgenic research.

The EH-2 transgenics did not show any significant increase in sinigrin content over that of the wild type EH-2, although there is accumulation of transcript of the transgene (Fig 6B). EH-2 is a low GS line (~12 μmol g^-1^ DW of seed) having mutated *MYB28*, that controls the basal level synthesis of aliphatic GS [19,20]. Transgenic lines of EH-2, therefore, have limited availability of the substrates for GS core structure formation and modification and hence, remain low in sinigrin despite accumulation of *BjuB*.*CYP79F1* transcripts. Such uncoupling of GS metabolite levels from the level of transcript accumulation for aliphatic GS biosynthetic genes has been shown in *A*. *thaliana* [36]. It has also been demonstrated that expression of most of the genes involved in aliphatic GS biosynthesis is repressed in Arabidopsis plants in which *MYB28* was knocked out [37,38]. This fact has been put to practice in *B*. *juncea* by down regulating *MYB28* through transgenic approach to develop low GS lines [39]. This corroborates with our observation on EH-2 transgenic lines.

Our results also lend credence to the earlier reports from *Arabidopsis* [31,40] regarding the involvement of the gene *CYP79F1* (At1g16410) in modulating the level of short-chain methionine-derived aliphatic GS. Hansen et al. [40] reported that when *CYP79F1* of Arabidopsis was heterologously expressed in *E*. *coli*, there was conversion of dihomo- and trihomomethionine to their corresponding aldoximes. In the same year, an Arabidopsis mutant *bus1-1f* that contained an En-1 insertion in the gene *CYP79F1* revealed a complete lack of short-chain methionine-derived GS. Conversely, the overexpression of *CYP79F1* in Arabidopsis led to an increased level of short-chain methionine-derived GS [31].

It was inferred from the present study on *B*. *juncea* and the sequence analyses of other *Brassica* species, that *CYP79F2* is absent in all these cases. It has been demonstrated in *A*. *thaliana* that *CYP79F1* catalyses synthesis of both short and long chain aliphatic GS, while *CYP79F2* catalyses that of only long chain aliphatic GS [40,41]. Therefore the absence of *CYP79F2* in *Brassica* species corresponds well with the fact that all profiles of aliphatic GS in brassicas are composed of short chain GS [27].

Some recent genomic studies provide indirect evidence in support of our findings. Comparative genome analysis using genome survey sequences identified non-synonymous SNPs in *CYP79F1* between a sinigrin rich *B*. *juncea* var. *tumida* and a *B*. *rapa* line free of sinigrin [42]. The *CYP79F1* expression was found to be upregulated in leaves of *B*. *juncea* as compared to *B*. *rapa*, suggesting difference in aliphatic GS content between *B*. *juncea* and *B*. *rapa*. However, it was not clear from the study whether the comparison of *CYP79F1* of *B*. *rapa* was with *CYP79F1* of the A or B genome of *B*. *juncea*. RNA-seq analysis of transcriptome and GS metabolism in a broccoli (*B*. *oleracea var*. *italica*) line rich in 4C GS could not detect orthologs for both *CYP79F1* and *CYP79F2* [43].

The present study resolved the genetic control of 3C GS and identified *BjuB*.*CYP79F1* located in the LG B4 of B genome as the candidate gene responsible for the 3C GS biosynthesis in *B*. *juncea*. *B*. *nigra* (BB) is the provider of B genome in allopolyploid *B*. *juncea* (AABB) and contributes a functional 3C GS pathway to *B*. *juncea* because sinigrin is the predominant aliphatic GS fraction in *B*. *nigra*. On the other hand, the A genome contributor *B*. *rapa* (AA) does not synthesize sinigrin and hence does not contribute to sinigrin biosynthesis in *B*. *juncea*. The fact was earlier confirmed by QTL analysis by Ramchiary et al. [18] where 3C GS QTL was detected only in the B genome (*J16Gsl4* in LG B4) and none in the A genome. Moreover, the observation of restoration of the phenotype in transgenic lines of NIL *J16Gsl4* in the present study not only identified the *CYP79F1* as the candidate gene for sinigrin biosynthesis in *B*. *juncea* but also revealed the fact that genes upstream and downstream to *CYP79F1* in aliphatic GS biosynthesis are functional in NIL *J16Gsl4*. It could be one of the major reasons for not being able to map a B genome paralogue of *GSL-PRO* (a member of the MAM gene family involved in the side chain elongation of aliphatic GS and is an upstream gene to *CYP79F1*) to the NIL *J16Gsl4* QTL region [19]. On the contrary, *GSL-PRO* has been proposed as the candidate gene controlling sinigrin biosynthesis in *B*. *oleracea* [15–17]. This points to the possible existence of two types of controls for 3C GS biosynthesis in *Brassica* species, one that operates at the chain elongation level in the C genome and the other that works at core GS structure formation level in the B genome.

The study also indicates that *BjuB*.*CYP79F1* in all probability is involved in conversion of methionine substrate (homomethionine) to aldoxime in the 3C GS biosynthesis pathway. If so, it puts forth the obvious question—are there specific *CYP79F1* homologs that are involved in the 4C and 5C pathways in *B*. *juncea*? A detailed molecular analysis of A genome specific *CYP79F1* which has been shown to be functional from the transcriptome data of *B*. *rapa* [44] and *B*. *juncea* [34] might throw some light on the specificity of this homolog.

Natural germplasm of *B*. *juncea* can be classified into two chemo-types for aliphatic GS [5]. Depending on the end use of the product, different artificial selection pressures have been applied for the genetic improvement of this crop. In India, mustard is grown for edible oil purpose; hence yield components and oil content enhancement traits have been the focus for selection and no selection was done for GS types. Thereby all the Indian germplasm contain both 3C (sinigrin) and 4C (gluconapin) types of GS. On the other hand, in the western hemisphere, only east European type mustard is grown which is used as leafy vegetable and condiment and lines with beneficial sinigrin fraction was mainly selected. Hence from the breeding point of view, the candidate gene polymorphism in *BjuB*.*CYP79F1* elaborated in the present study in *B*. *juncea* can be used as functional foreground selection marker for the efficient transfer of the trait (either high or low allele for sinigrin, as required) through marker-assisted breeding.

## Supporting Information

S1 Fig PCR amplification banding pattern of 15 genes of aliphatics GS pathway from *B*. *juncea* lines Varuna (V) and Heera (H); *B*. *rapa* (R) and *B*. *nigra* (N). (DOCX)

S2 Fig Nucleotide alignment of the genomic sequences of *CYP79F1s* from various Brassicaceae species. (DOCX)

S3 Fig Amino acid sequence alignment of *CYP79F1s* showing different conserved motifs from various species of Brassicaceae. (DOCX)

S4 Fig Neighbour joining tree constructed from *CYP79F1* CDS sequences of few species of Brassicaceae. (DOCX)

S5 Fig A scheme for the development of F_2_ and F_3_ populations derived from a cross between Varuna and QTL-NIL *J16Gsl4*. (DOCX)

S6 Fig Phenotype(3C GS)-genotype(*BjuB*.*CYP79F1*) co-segregation graph of 95 F_2_ segregants derived from Varuna x QTL-NIL *J16Gsl4*. (DOCX)

S1 Table List of the primers used for amplification of the GS biosynthetic genes from 1g and 2g chromosomes of *A*. *thaliana*. (DOCX)

S2 Table List of the B genome specific nested primers designed for sequencing and genome walk. (DOCX)

S3 Table List and properties of genes from the sequence of BAC clone from *B*. *juncea* line Heera and their corresponding orthologs in *A*. *thaliana* and *B*. *rapa*. (DOCX)

S4 Table Comparative data on genotype for the gene *BjuB*.*CYP79F1*, aliphatic GS profile and mRNA expression of 100 F2 segregants derived from the cross Varuna x QTL-NIL *J16Gsl4*. (DOCX)

S5 Table Seed aliphatic GS profile from T_1_ and T_2_ seeds of five high expressers of sinigrin from QTL-NIL *J16Gsl4* and EH-2 containing transgene 35S:*BjuB*.*CYP79F1*. (DOCX)

## Abbreviations

μmol g^-1^: Micromoles per gram
3C: Three-carbon
4C: Four-carbon
5C: Five-carbon
cDNA: Complementary deoxyribonucleic acid
cM: CentiMorgan
cv: Cultivar
*CYP79F1*: Cytochrome P450 monooxygenase 79F1
*CYP79F2*: Cytochrome P450 monooxygenase 79F2
DW: Dry weight
ER: Endoplasmic reticulum
GS: Glucosinolates
LG: Linkage group
mRNA: Messenger ribonucleic acid
NIL: Near-isogenic line
QTL: Quantitative trait locus
RT-PCR: Reverse transcriptase polymerase chain reaction
TE: Transposable element
