# Supplementary material for: BjuB.CYP79F1 Regulates Synthesis of Propyl Fraction of Aliphatic Glucosinolates in Oilseed Mustard Brassica juncea: Functional Validation through Genetic and Transgenic Approaches
Source: PLoS One. 2016 Feb 26;11(2):e0150060. doi: 10.1371/journal.pone.0150060 (PMC4769297; doi:10.1371/journal.pone.0150060)
Supplement: S1 Fig — (DOCX) [file pone.0150060.s001.docx]

**S1 Fig:** PCR amplification banding pattern of 15 genes of aliphatics GS pathway from *B. juncea* lines Varuna (V) and Heera (H); *B. rapa* (R) and *B. nigra* (N). L is 100 bp ladder (NEB). Gene IDs have been shown below the gel pictures.
