# Supplementary material for: BjuB.CYP79F1 Regulates Synthesis of Propyl Fraction of Aliphatic Glucosinolates in Oilseed Mustard Brassica juncea: Functional Validation through Genetic and Transgenic Approaches
Source: PLoS One. 2016 Feb 26;11(2):e0150060. doi: 10.1371/journal.pone.0150060 (PMC4769297; doi:10.1371/journal.pone.0150060)
Supplement: S2 Fig — (DOCX) [file pone.0150060.s002.docx]

**S2 Fig:** Nucleotide alignment of the genomic sequences of *CYP79F1s* from the analysed Brassicaceae species: *A. thaliana* (At1g16400 and At1g16410), *B. rapa* (Bra026058), *B. oleracea* (Bol038222), *B. napus* A genome genes GSBRNA2T00057963001 (A3001) and GSBRNA2T00057964001 (A4001), *B. napus* C genome gene GSBRNA2T00054164001 (C4001), *B. nigra* (*BniB.CYP79F1*) and *B. juncea* cvs. Varuna [*BjuB.CYP79F1*(V)] and Heera [*BjuB.CYP79F1*(H)]. ClustalW alignment was performed using MegAlign software package of DNASTAR Inc. Positions of degenerate primers have been shown using arrows above the sequence. Positions of B genome specific primers have been shown using arrows below the sequence.
