# Supplementary material for: BjuB.CYP79F1 Regulates Synthesis of Propyl Fraction of Aliphatic Glucosinolates in Oilseed Mustard Brassica juncea: Functional Validation through Genetic and Transgenic Approaches
Source: PLoS One. 2016 Feb 26;11(2):e0150060. doi: 10.1371/journal.pone.0150060 (PMC4769297; doi:10.1371/journal.pone.0150060)
Supplement: S3 Fig — (DOCX) [file pone.0150060.s003.docx]

**S3 Fig:** Amino acid sequence alignment of *CYP79F1s* showing different conserved motifs from the analysed Brassicaceae species: *A. thaliana* (At1g16400 and At1g16410), *B. rapa* (Bra026058), *B. oleracea* (Bol038222), *B. napus* A genome (GSBRNA2T00057963001 and GSBRNA2T00057964001), *B. napus* C genome (GSBRNA2T00054164001), *B. nigra* (BniB.CYP79F1) and *B. juncea* cv. Varuna (BjuB.CYP79F1 V). ClustalW alignment was performed using MegAlign software package of DNASTAR Inc.
