# Supplementary material for: BjuB.CYP79F1 Regulates Synthesis of Propyl Fraction of Aliphatic Glucosinolates in Oilseed Mustard Brassica juncea: Functional Validation through Genetic and Transgenic Approaches
Source: PLoS One. 2016 Feb 26;11(2):e0150060. doi: 10.1371/journal.pone.0150060 (PMC4769297; doi:10.1371/journal.pone.0150060)
Supplement: S4 Fig — (DOCX) [file pone.0150060.s004.docx]

**
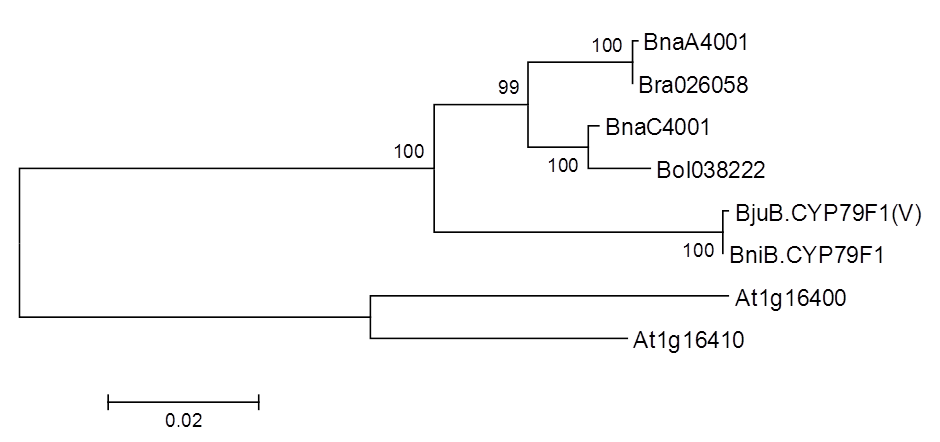
**

**S4 Fig:** Neighbour joining tree constructed from *CYP79F1* CDS sequences of few Brassicaceae species, i.e. *A. thaliana* [*CYP79F2* (At1g16400) and *CYP79F1* (At1g16410)], *B. rapa* (Bra026058), *B. oleracea* (Bol038222), *B. napus* A genome (BnaA4001; gene ID GSBRNA2T00057964001), *B. napus* C genome (BnaC4001; gene ID GSBRNA2T00054164001), *B. nigra* (*BniB.CYP79F1*) and *B. juncea* cv. Varuna [*BjuB.CYP79F1*(V)] using MEGA5. The CDS sequences of *BniB.CYP79F1* and *BjuB.CYP79F1*(V) were obtained from the sequences submitted in the National Center for Biotechnology Information GenBank database (accession numbers KT254223 and KT254222, respectively). The CDS sequences for the rest of Brassicaceae species were obtained from BRAD database (http://brassicadb.org/brad/). The scale indicates number of base substitutions per site. The numbers at the nodes indicate the percentages of bootstrap support after 1000 replicates.
