# Supplementary material for: BjuB.CYP79F1 Regulates Synthesis of Propyl Fraction of Aliphatic Glucosinolates in Oilseed Mustard Brassica juncea: Functional Validation through Genetic and Transgenic Approaches
Source: PLoS One. 2016 Feb 26;11(2):e0150060. doi: 10.1371/journal.pone.0150060 (PMC4769297; doi:10.1371/journal.pone.0150060)
Supplement: S5 Fig — (DOCX) [file pone.0150060.s005.docx]

**S5 Fig:** A scheme for the development of F_2_ and F_3_ populations derived from a cross between Varuna and QTL-NIL *J16Gsl4*.
