# Supplementary material for: BjuB.CYP79F1 Regulates Synthesis of Propyl Fraction of Aliphatic Glucosinolates in Oilseed Mustard Brassica juncea: Functional Validation through Genetic and Transgenic Approaches
Source: PLoS One. 2016 Feb 26;11(2):e0150060. doi: 10.1371/journal.pone.0150060 (PMC4769297; doi:10.1371/journal.pone.0150060)
Supplement: S6 Fig — (DOCX) [file pone.0150060.s006.docx]

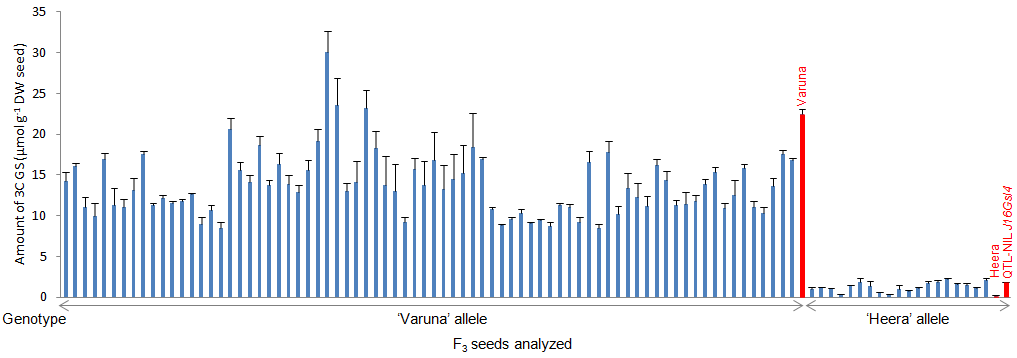


**S6 Fig:** Phenotype-genotype co-segregation graph of 95 F_2_ segregants derived from Varuna x QTL-NIL *J16Gsl4*. Amount of 3C GS in seeds of parental lines Varuna, Heera and QTL-NIL *J16Gsl4* lines has been shown in red bars. Values are given as mean of two replicates.
