# Supplementary material for: BjuB.CYP79F1 Regulates Synthesis of Propyl Fraction of Aliphatic Glucosinolates in Oilseed Mustard Brassica juncea: Functional Validation through Genetic and Transgenic Approaches
Source: PLoS One. 2016 Feb 26;11(2):e0150060. doi: 10.1371/journal.pone.0150060 (PMC4769297; doi:10.1371/journal.pone.0150060)
Supplement: S1 Table — (DOCX) [file pone.0150060.s007.docx]

**S1 Table:** List of the primers used for amplification of the GS biosynthetic genes from 1g and 2g chromosomes encompassing the syntenous region of QTL *J16Gsl4* in *B. juncea*.

| **Locus** | | **Primer code** | **Sequence (5'-3')** | **Expected amplicon size (bp) *** | **Observed amplicon size (bp) **** |
| --- | --- | --- | --- | --- | --- |
| ***A. thaliana*** | ***B. rapa*** |  |  |  |  |
| At1g16400 (CYP79F2)  At1g16410- Tandem (CYP79F1) | Bra026058 | GS1- FP  GS1- RP  1g125-FP  1g125-RP | GACGAGATCGCTMGAGAAG  CTCAACRGACAARWGAAGAGGY  CATGAAGATGAAAARAGTGATCA  ATCATRATYGTCCCGACTT | 1844  1592 | ~1700  ~1500 |
| At1g78370 (GSTU20)  At1g78360- Tandem (GSTU21)  At1g78380- Tandem (GSTU19) | Bra008357  Bra003644- Tandem | GS13- FP  GS13- RP | GCCWAGYATGTTCGGSATGAGG  CGATGCTGAAGTTACCAAAC | 1029 | ~800, ~700, ~650, ~400 |
| At2g20610 (SUR1) | Bra036490  Bra031132  Bra036703 | GS4- FP  GS4- RP | CTTGCTTCCGYACCTGTATCG  GAGTATCTTGTTCTTCTTTGCAAAG | 1211 | ~1300, ~1200 |
| At1g24100 (UGT74B1) | Bra024634 | GS10- FP  GS10- RP | CTTACCCAGTCCAAGGCCAC  GATCAGAGCTTCCTCCTTCACTC | 1457 | ~1350, ~600 |
| At2g31790 (UGT74C1) | Bra005641  Bra021743 | GS6- FP  GS6- RP | CCACACMATCYACGACGGTTTC  GCTGAGGAACCCACTTAGC | 1974 | ~1600, ~1300, ~600 |
| At1g74090 (SOT18)  At1g74100- Tandem (SOT16) | Bra015938- Tandem  Bra008132  Bra003817- Tandem | GS11- FP  GS11- RP  GS12- FP  GS12- RP | CGYTYMCWMASRRSAAAGGCTGGAG  AGCCATCTCCGGAGTCAG  GACTTCCTCGTCTGYAGCTACCC  GAGCAGCCATCTCYGGAGTC | 859  738 | ~850  ~750 |
| At1g18590 (SOT17) | Bra025668 | At1g-420F  At1g-420R | GAGAAGAACCAGAAACACTACCAAG  MCCCATAAACTCAGCYAAYCTCTTC | 695 | ~700 |
| At1g65860 (GS-OX1) |  | GS17- FP  GS17- RP | GATAAAATCATGGCCAGGAAAG  CTACTAATATCGGCACCGCTC | 1221 | No amplification |
| At1g62540 (GS-OX2)  At1g62560- Tandem (GS-OX3)  At1g62570- Tandem (GS-OX4) |  | GS14- FP  GS14- RP  GS15- FP  GS15- RP  GS16- FP  GS16- RP | CAACCTCCCGAGAGAGTG  GGCTATGGATCTGCTTTCC  GCGGTTCATGCAGATACTG  CTGATTTGGGGAAATAAGAAGG  GTCWAGAGACYCGAGAAGGTATCC  GCAATGCACAATRGTATCAGC | 1406  1125  1444 | ~1400, ~1200, ~500, ~400  ~1100  ~1000, ~900 |
| At1g12200  (Putative FMO) | Bra019747  Bra026986 | GS2- FP  GS2- RP | GTGAGCTACGTCGTGAAGG  CATGCTTATACAACGGTCCAAC | 1674 | ~1550, ~1500 |

* Expected amplicon size is based on *B. rapa* and wherever, there is no homolog of a gene in *B. rapa*, the expected size is based on Arabidopsis.

** Observed amplicon size is in *B. juncea*.
