# Supplementary material for: BjuB.CYP79F1 Regulates Synthesis of Propyl Fraction of Aliphatic Glucosinolates in Oilseed Mustard Brassica juncea: Functional Validation through Genetic and Transgenic Approaches
Source: PLoS One. 2016 Feb 26;11(2):e0150060. doi: 10.1371/journal.pone.0150060 (PMC4769297; doi:10.1371/journal.pone.0150060)
Supplement: S2 Table — (DOCX) [file pone.0150060.s008.docx]

**S2 Table:** List of the B genome specific nested primers designed for sequencing and genome walk of genes *BjuB.CYP79F1* and *BniB.CYP79F1* from *B. juncea* and *B. nigra*, respectively.

| **Primer code** | **Primer Sequence (5’ – 3’)** |
| --- | --- |
| **Primers for sequencing** | |
| GS1B-NS-FP | CGCAGACCGGCCAAATAC |
| GS1D-NS-FP | CACCGGTGAACAGTTCATGAAA |
| GS1E-NS-FP | CGACGTTAGAGAGATCTCGAGGAA |
| GS1F-NS-FP | GGCTGGAACGTTGATGGTCT |
| GS1G-NS-FP | ACGAGAGGGTGGAGTTATGGAGA |
| GS1C-NS-FP | GTTGCAGAGAAACATTCAGGG |
| GS1B-NS-RP | AAATAACCTCCGAGGGTGC |
| GS1C-NS-RP | GTGGCTACCTGTCCAAAACA |
| GS1D-NS-RP | GAGAAAGCTCCTTCGAGATTCCATT |
| GS1E-NS-RP | CATCATGATCGTCCCGACTTTC |
| **Primers for genome walk** | |
| **5’ genome walk** | |
| 5’-GSP-R1 | TTCCTCGAGATCTCTCTAACGTCGGCT |
| 5’-GSP-R2 | AGCAAGGAGGTTGTCCGCTTCAATGT |
| 5’GSP-B-R1 | GCGATCTCCGGTTTTTGCCCTTTCGT |
| 5’GSP-B-R2 | TATTCGGCCAAGCAAAGTGATGGATGCT |
| **3’ genome walk** | |
| 3’-GSP-F1 | CTCCACCAGTAGTTTTGTATGATGCGTG |
| 3’-GSP-F2 | TTGGACAGGTAGCCACATTCACGTCGC |
