# Supplementary material for: BjuB.CYP79F1 Regulates Synthesis of Propyl Fraction of Aliphatic Glucosinolates in Oilseed Mustard Brassica juncea: Functional Validation through Genetic and Transgenic Approaches
Source: PLoS One. 2016 Feb 26;11(2):e0150060. doi: 10.1371/journal.pone.0150060 (PMC4769297; doi:10.1371/journal.pone.0150060)
Supplement: S3 Table — (DOCX) [file pone.0150060.s009.docx]

**S3 Table:** List and properties of genes from the sequence of BAC clone from *B. juncea* line Heera and their corresponding orthologs in *A. thaliana* and *B. rapa*.

| ***B. juncea* BAC B 113 ‘B’ G12** | | | | | **Orthologous gene ID** | | **Description** |
| --- | --- | --- | --- | --- | --- | --- | --- |
| **Gene No.** | **Position** | | **Length (bp)** | **Spacer (bp)** | ***A. thaliana*** | ***B. rapa*** |  |
|  | **Begin** | **End** |  |  |  |  |  |
| **Contig1** | | | | | | | |
| Bj1 | 40760 | 41810 | 1050 | 917 | At3g23730 | Bra014975 | Xyloglucan endotransglucosylase / hydrolase |
| Bj2 | 37143 | 39843 | 2700 | 10519 | At3g23740 | Bra014976 | Unknown protein |
| Bj3 | 23487 | 26624 | 3137 | 907 | At3g23750 | Bra014977 | Leucine-rich repeat protein kinase family protein |
| Bj4 | 21576 | 22580 | 1004 | 770 | At3g43320 | - | Transposable element gene |
| Bj5 | 17621 | 20806 | 3185 | 913 | At4g11710 | - | Transposable element gene |
| Bj6 | 15608 | 16708 | 1100 | 846 | At3g23760 | Bra014978 | Unknown protein |
| Bj7 | 13974 | 14762 | 788 | 421 | At3g23770 | Bra014979 | Glycosyl hydrolase family 17 protein |
| Bj8 | 11221 | 13553 | 2332 | 231 | At1g16460 | Bra026055 | Mercaptopyruvate sulfurtransferase 2 |
| Bj9 | 10235 | 10990 | 755 | 604 | - | BAC KBrB057E05 | Unknown protein |
| Bj10 | 8203 | 9631 | 1428 | 852 | At1g16460 | Bra026056 | Root hair specific 3 (RSH3); kinase |
| Bj11 | 6537 | 7351 | 814 | 1539 | At1g16430 | Bra026057 | Surfeit locus protein 5 (SUR5) family protein |
| Bj12 | 4080 | 4998 | 918 | 923 | At2g21660 | Bra031210 | ATGRP7, GR-RBP7, CCR2 (Circadian rhythm and RNA Binding 2) |
| Bj13 | 2720 | 3157 | 437 | 1440 | At4g38680 | - | CSDP2, ATCSP2, GRP2 (Glycine rich protein 2) |
| Bj14 | 1089 | 1280 | 191 | 1088 | - | - | Unknown protein |
| **Contig2** | | | | | | | |
| Bj15 | 345 | 533 | 188 | 344 | - | - | Unknown protein |
| Bj16 | 549 | 1185 | 636 | 16 | - | - | Unknown protein |
| Bj17 | 1824 | 2075 | 251 | 639 | - | - | Unknown protein |
| Bj18 | 2222 | 2485 | 263 | 147 | - | - | Unknown protein |
| Bj19 | 2648 | 2866 | 218 | 163 | - | - | Unknown protein |
| Bj20 | 3467 | 3894 | 427 | 601 | - | - | Unknown protein |
| **Bj21 (*BjuB.CYP79F1*)** | **4699** | **5547** | **848** | **805** | **At1g16410** | **Bra026058** | ***CYP79F1*, oxidoreductase** |
| Bj22 | 7720 | 14120 | 6400 | 2173 | At1g16380 | Bra026059 | ATCHX1 (Cation exchanger 1); member of putative Na^+^/H^+^ antiporter family |
| Bj23 | 14266 | 15805 | 1539 | 146 | At1g16370 | Bra026060 | Organic cation / Carnitine transporter 6 (OCT6) |
| Bj24 | 20391 | 21911 | 1520 | 4586 | At1g16370 | Bra026061 |  |
| Bj25 | 25003 | 27198 | 2195 | 3092 | At4g23160 | - | Cysteine-rich receptor-like protein kinase (CRK8) |
| Bj26 | 27352 | 29385 | 2033 | 154 | At1g21280 | Bra025885 | Unknown protein |
| Bj27 | 31772 | 32008 | 236 | 2387 | - | - | Unknown protein |
| Bj28 | 33602 | 33957 | 355 | 1594 | At1g16370 | Bra026062 | Organic cation / Carnitine transporter 6 (OCT6) |
| **Contig3** | | | | | | | |
| Bj28 | 11857 | 13057 | 1200 | 10328 | At1g16370 | Bra026062 | Organic cation / Carnitine transporter 6 (OCT6) |
| Bj29 | 21 | 1529 | 1508 | 20 | At1g16350 | Bra026064 | Aldolase-type TIM barrel family protein |
| **Contig5** | | | | | | | |
| Bj29 | 44 | 532 | 488 | 43 | At1g16350 | Bra026064 | Aldolase-type TIM barrel family protein |
| Bj30 | 1046 | 4933 | 3887 | 514 | At1g16330 | Bra026065 | CYCB3;1 – Cyclin-dependent protein kinase |

Length – Total length (bp) of gene.

Spacer – Spacer length (bp) between the previous gene and the listed gene in the same contig
