# Supplementary material for: BjuB.CYP79F1 Regulates Synthesis of Propyl Fraction of Aliphatic Glucosinolates in Oilseed Mustard Brassica juncea: Functional Validation through Genetic and Transgenic Approaches
Source: PLoS One. 2016 Feb 26;11(2):e0150060. doi: 10.1371/journal.pone.0150060 (PMC4769297; doi:10.1371/journal.pone.0150060)
Supplement: S4 Table — (DOCX) [file pone.0150060.s010.docx]

**S4 Table:** Comparative data on genotype for the gene *BjuB.CYP79F1,* aliphatic GS profile and mRNA expression of 100 F_2_ segregants derived from the cross Varuna x QTL-NIL *J16Gsl4*.

| Plant Name | **Genotype** | **mRNA expression** | **Aliphatic GS (µmol g^-1^ DW in seed)** | | | | | |
| --- | --- | --- | --- | --- | --- | --- | --- | --- |
|  |  |  | **SIN** | | **GNA** | | **GBN** | **Total GS** |
| Varuna | a | a | 22.81 | | 103.04 | | 1 | 126.85 |
| Heera | b | n.a. | 0.09 | | 10.02 | | 0.18 | 10.29 |
| QTL-NIL *J16Gsl4* | b | b (no expression) | 1.73 | | 118.55 | | 1.61 | 121.89 |
| 1 | a | a | 13.53 | | 137.28 | | 1.36 | 152.17 |
| 2 | a | a | 15.89 | | 135.52 | | 1.43 | 152.84 |
| 3 | a | n.a. | 10.24 | | 136.51 | | 1.59 | 148.34 |
| 4 | a | n.a. | 9.14 | | 127.7 | | 1.53 | 138.37 |
| 5 | a | n.a. | 16.52 | | 127.48 | | 1.4 | 145.4 |
| 6 | a | n.a. | 10.16 | | 139.95 | | 1.59 | 151.7 |
| 7 | b | b | 0 | | 142.22 | | 2.19 | 144.41 |
| 8 | a | n.a. | 10.58 | | 127.07 | | 1.55 | 139.2 |
| 9 | a | n.a. | 12.33 | | 144 | | 1.72 | 158.05 |
| 10 | a | n.a. | 17.78 | | 135.33 | | 1.76 | 154.87 |
| 11 | a | n.a. | 11.22 | | 126.89 | | 1.58 | 139.69 |
| 12 | a | n.a. | 11.94 | | 122.26 | | 1.52 | 135.72 |
| 13 | a | n.a. | 11.39 | | 132.66 | | 1.63 | 145.68 |
| 14 | a | n.a. | 11.96 | | 141.39 | | 1.78 | 155.13 |
| 15 | a | n.a. | 12.62 | | 137.92 | | 1.56 | 152.1 |
| 16 | a | n.a. | 8.37 | | 115.31 | | 1.72 | 125.4 |
| 17 | a | n.a. | 11.07 | | 129.86 | | 1.88 | 142.81 |
| 18 | a | n.a. | 7.95 | | 115 | | 1.84 | 124.79 |
| 19 | b | b | 0 | | 134.3 | | 1.11 | 135.41 |
| 20 | b | b | no data | | | | | |
| 21 | a | n.a. | 21.45 | 100.6 | | 1.83 | | 123.88 |
| 22 | a | n.a. | 15.12 | 99.29 | | 0.61 | | 115.02 |
| 23 | a | n.a. | 13.59 | 93.07 | | 2.23 | | 108.89 |
| 24 | a | n.a. | 18.11 | 89.15 | | 1.96 | | 109.22 |
| 25 | a | n.a. | 13.42 | 96.33 | | 1.97 | | 111.72 |
| 26 | b | n.a. | no data | | | | | |
| 27 | b | b | 0 | 123.04 | | 0.98 | | 124.02 |
| 28 | a | n.a. | 16.27 | 114.06 | | 1.63 | | 131.96 |
| 29 | a | n.a. | 12.33 | 94.34 | | 1.45 | | 108.12 |
| 30 | a | n.a. | 13.42 | 117.05 | | 0.99 | | 131.46 |
| 31 | b | n.a. | 0 | 132.44 | | 2.14 | | 134.58 |
| 32 | b | n.a. | 0 | 147.18 | | 4.47 | | 151.65 |
| 33 | b | n.a. | 0 | 144.23 | | 1.23 | | 145.46 |
| 34 | a | n.a. | 16.2 | 123.32 | | 1.09 | | 140.61 |
| 35 | b | n.a. | 0 | 156.98 | | 1.22 | | 158.2 |
| 36 | a | n.a. | 19.66 | 112.06 | | 1 | | 132.72 |
| 37 | b | n.a. | no data | | | | | |
| 38 | a | a | 31.57 | 127.86 | | 1.17 | | 160.6 |
| 39 | a | a | 23.02 | 115.43 | | 2.08 | | 140.53 |
| 40 | a | a | 12.56 | 93.88 | | 0.74 | | 107.18 |
| 41 | a | n.a. | 12.56 | 95.97 | | 1.35 | | 109.88 |
| 42 | a | n.a. | 24.25 | 149.76 | | 1.91 | | 175.92 |
| 43 | a | n.a. | 18.58 | 103.61 | | 0.87 | | 123.06 |
| 44 | a | n.a. | 12.21 | 104.67 | | 1.28 | | 118.16 |
| 45 | a | n.a. | 11.57 | 99.66 | | 1.34 | | 112.57 |
| 46 | a | n.a. | 9.22 | 89.95 | | 1.16 | | 100.33 |
| 47 | b | n.a. | 0 | 136.43 | | 1.29 | | 137.72 |
| 48 | a | n.a. | 16.27 | 112.12 | | 1.04 | | 129.43 |
| 49 | b | n.a. | 0 | 136.83 | | 1.45 | | 138.28 |
| 50 | b | n.a. | 0 | 136.73 | | 1.62 | | 138.35 |
| 51 | a | n.a. | 15.33 | 104.93 | | 1.02 | | 121.28 |
| 52 | a | n.a. | 18.65 | 118.62 | | 1.38 | | 138.65 |
| 53 | b | n.a. | 0 | 116.5 | | 1.26 | | 117.76 |
| 54 | b | n.a. | 0 | 127.93 | | 1.21 | | 129.14 |
| 55 | a | n.a. | 13.18 | 119.29 | | 1.11 | | 133.58 |
| 56 | a | n.a. | 13.61 | 117.82 | | 1.61 | | 133.04 |
| 57 | a | n.a. | 14.34 | 123.61 | | 1.47 | | 139.42 |
| 58 | a | n.a. | 17.15 | 110.01 | | 1.29 | | 128.45 |
| 59 | a | n.a. | 16.95 | 116.63 | | 1.11 | | 134.69 |
| 60 | b | n.a. | 0 | 128.54 | | 1.8 | | 130.34 |
| 61 | a | n.a. | 10.93 | 131.42 | | 1.84 | | 144.19 |
| 62 | a | n.a. | 8.66 | 113.94 | | 1.65 | | 124.25 |
| 63 | a | n.a. | 9.72 | 119.26 | | 1.82 | | 130.8 |
| 64 | a | n.a. | 10.6 | 114.6 | | 1.81 | | 127.01 |
| 65 | a | n.a. | 9.16 | 106.9 | | 1.7 | | 117.76 |
| 66 | a | n.a. | 9.35 | 119.9 | | 1.72 | | 130.97 |
| 67 | a | n.a. | 8.39 | 131.96 | | 1.93 | | 142.28 |
| 68 | a | n.a. | 11.42 | 120.46 | | 2.2 | | 134.08 |
| 69 | b | n.a. | 0 | 132.43 | | 1.71 | | 134.14 |
| 70 | a | n.a. | 11.25 | 127 | | 2.1 | | 140.35 |
| 71 | a | n.a. | 8.84 | 117.69 | | 2.04 | | 128.57 |
| 72 | b | n.a. | 0 | 122.3 | | 2.01 | | 124.31 |
| 73 | a | n.a. | 15.75 | 109.37 | | 1.56 | | 126.68 |
| 74 | a | n.a. | 8.15 | 107.28 | | 1.79 | | 117.22 |
| 75 | b | n.a. | 0 | 144.9 | | 1.75 | | 146.65 |
| 76 | a | n.a. | 17.01 | 127.79 | | 2.25 | | 147.05 |
| 77 | a | n.a. | 9.63 | 102.4 | | 1.33 | | 113.36 |
| 78 | b | n.a. | 0 | 129.11 | | 1.75 | | 130.86 |
| 79 | a | n.a. | 12.32 | 138.53 | | 1.91 | | 152.76 |
| 80 | a | n.a. | 11.26 | 137.95 | | 1.97 | | 151.18 |
| 81 | a | n.a. | 11.12 | 117.64 | | 1.51 | | 130.27 |
| 82 | a | n.a. | 16.16 | 119.76 | | 1.73 | | 137.65 |
| 83 | a | n.a. | 14.17 | 110.83 | | 1.74 | | 126.74 |
| 84 | a | n.a. | 11.22 | 132.4 | | 1.76 | | 145.38 |
| 85 | a | n.a. | 11.46 | 145.72 | | 1.97 | | 159.15 |
| 86 | a | n.a. | 11.81 | 127.12 | | 1.58 | | 140.51 |
| 87 | b | n.a. | 0 | 147.31 | | 2.27 | | 149.58 |
| 88 | a | n.a. | 13.93 | 111.19 | | 1.69 | | 126.81 |
| 89 | a | n.a. | 15.21 | 124.6 | | 1.49 | | 141.3 |
| 90 | a | n.a. | 10.85 | 128.41 | | 1.73 | | 140.99 |
| 91 | a | n.a. | 12.51 | 144.57 | | 2.27 | | 159.35 |
| 92 | a | n.a. | 15.78 | 134.21 | | 2.15 | | 152.14 |
| 93 | a | n.a. | 10.99 | 139.67 | | 1.87 | | 152.53 |
| 94 | a | n.a. | 10.22 | 126.63 | | 1.93 | | 138.78 |
| 95 | b | n.a. | 0 | 153.34 | | 2.14 | | 155.48 |
| 96 | b | n.a. | no data | | | | | |
| 97 | a | n.a. | 13.57 | 125.75 | | 2.04 | | 141.36 |
| 98 | b | n.a. | no data | | | | | |
| 99 | a | n.a. | 17.58 | 121.86 | | 1.38 | | 140.82 |
| 100 | a | n.a. | 16.77 | 111.43 | | 1.54 | | 129.74 |

Values are given as mean of 2 replicates. n.a. – not analyzed; DW – Dry weight; SIN – Desulfosinigrin; GNA – Desulfogluconapin; GBN – Desulfoglucobrassicanapin.
