# Supplementary material for: BjuB.CYP79F1 Regulates Synthesis of Propyl Fraction of Aliphatic Glucosinolates in Oilseed Mustard Brassica juncea: Functional Validation through Genetic and Transgenic Approaches
Source: PLoS One. 2016 Feb 26;11(2):e0150060. doi: 10.1371/journal.pone.0150060 (PMC4769297; doi:10.1371/journal.pone.0150060)
Supplement: S5 Table — (DOCX) [file pone.0150060.s011.docx]

**S5 Table:** Seed aliphatic GS profile from T_1_ and T_2_ seeds of five high expressers of sinigrin from QTL-NIL *J16Gsl4* and EH-2 containing transgene 35S:*BjuB.CYP79F1*.

| **Plant Name** | **Aliphatic GS (µmol g^-1^ DW)** | | | |
| --- | --- | --- | --- | --- |
|  | **SIN** | **GNA** | **GBN** | **Total GSL** |
| Varuna (wild type) | 21.9 (14.8) | 124.9 (150.5) | 1.5 (2.1) | 149.6 (167.7) |
| QTL-NIL *J16Gsl4* transgenics | | | | |
| QTL-NIL *J16Gsl4* untransformed | 0.4 (0) | 198.5 (205.6) | 2.6 (2.9) | 202.7 (208.7) |
| 1 | 17.8 (13.3) | 197.2 (194.4) | 2.1 (2.6) | 217.6 (210.5) |
| 2 | 21.9 (18.8) | 163.7 (149) | 1.8 (2.6) | 188.4 (171.1) |
| 3 | 24.6 (18.6) | 157 (159.5) | 1.8 (2.2) | 185.1 (181) |
| 4 | 23.7 (18.9) | 148.1 (157.4) | 1.7 (2.4) | 175.4 (179.3) |
| 5 | 25.5 (18.2) | 144.7 (163.8) | 1.5 (2.1) | 173.3 (185) |
| EH-2 transgenics | | | | |
| EH-2 untransformed | 0.2 | 7.6 | 0.2 | 11.5 |
| 1 | 1.6 | 6.1 | 0.4 | 12 |
| 2 | 1.2 | 5.7 | 0.4 | 11.3 |
| 3 | 2.4 | 7.6 | 0.5 | 15.2 |
| 4 | 1.5 | 7.3 | 0.5 | 14 |
| 5 | 1.8 | 5.3 | 0.4 | 12.6 |

Values of T_1_ seeds are given as mean of four replicates. Values of T_2_ seeds (in parentheses) of QTL-NIL *J16Gsl4* transgenic lines are given as mean of two replicates.
